# Evidence against a Role for β-Arrestin1 in STAT1 Dephosphorylation and the Inhibition of Interferon-γ Signaling

**DOI:** 10.1016/j.molcel.2013.02.024

**Published:** 2013-04-11

**Authors:** Christin Pelzel, Andreas Begitt, Nikola Wenta, Uwe Vinkemeier

**Affiliations:** 1 The University of Nottingham, School of Biomedical Sciences, Nottingham NG7 2UH, UK

## Abstract

Signal transducer and activator of transcription 1 (STAT1) is activated by tyrosine phosphorylation upon interferon-γ (IFNγ) stimulation, which results in the expression of genes with antiproliferative and immunomodulatory functions. The inactivation of STAT1 occurs through tyrosine dephosphorylation by the tyrosine phosphatase TC45. It was proposed that recruitment of TC45 required the direct interaction of STAT1 with the scaffold protein β-arrestin1, making β-arrestin1 an essential negative regulator of STAT1 and IFNγ signaling (Mo et al., 2008). We tested the relevance of β-arrestin1 for STAT1 activity. Our results do not confirm β-arrestin1 as a STAT1-interacting protein. The STAT1 phosphorylation/dephosphorylation cycle was found to be unaffected by both the overexpression and the genetic deletion of β-arrestin1. Accordingly, β-arrestin1 did not inhibit STAT1 transcriptional activity or the induction of IFNγ target genes in response to IFNγ. Our data indicate that β-arrestin1 is dispensable for STAT1 dephosphorylation and the termination of IFNγ signaling.

## Highlights

► Contrary to the findings of Mo et al. (2008), β-arrestin1 does not inhibit IFNγ signaling ► β-arrestin1 is dispensable for STAT1 dephosphorylation ► β-arrestin1 does not suppress STAT1 target genes expression ► β-arrestin1 is not confirmed as a STAT1-interacting protein

## Introduction

The cytokine interferon-γ is critical for protection against viral and bacterial infections and tumor development. Its biological activities require the phosphorylation of STAT1 at a single tyrosine residue (Stark and Darnell, 2012). This crucial event is also termed “STAT activation,” as it transforms the STAT1 dimers into DNA binding transcription factors. “STAT1 inactivation,” namely the enzymatic reversal of tyrosine phosphorylation, accordingly is equally important for physiological signaling (Liu et al., 2011). The tyrosine phosphatase TC45 is the major STAT1-inactivating enzyme (ten Hoeve et al., 2002). Understanding the biochemical and structural details of STAT1 dephosphorylation therefore is required for understanding the physiological regulation of IFNγ signaling as well as for the development of therapeutic STAT1 modulators, e.g., for viral and immune diseases (Borden et al., 2007). In the cell nucleus, STAT1 inactivation is ultimately limited by the kinetics of DNA binding, whereby STAT1 is available for dephosphorylation only in its DNA unbound state (Meyer et al., 2003). Recent results indicate that dephosphorylation is a multistep process that requires STAT1 dimers to undergo extensive spatial reorientation (Zhong et al., 2005; Mertens et al., 2006). Hydrodynamic modeling of analytical ultracentrifugation results obtained with purified STAT1 indicated moreover that the reorientation of the recombinant STAT1 dimers is considerably slower (t_1/2_ 20–40 min; Wenta et al., 2008) than the dephosphorylation of endogenous STAT1 in living cells (t_1/2_ <15 min; Haspel et al., 1996). In fact, the acetylation of two particular lysine residues of STAT1 was reported to enhance its dephosphorylation by facilitating recruitment of tyrosine phosphatase TC45 (Krämer et al., 2009), but this claim was subsequently invalidated (Antunes et al., 2011). Another posttranslational modification, namely SUMO conjugation, can enhance the dephosphorylation of STAT1 by increasing its solubility, yet SUMO does not itself partake in the actual dephosphorylation step (Droescher et al., 2011). The only STAT1-interacting protein known to directly enhance the dephosphorylation reaction thus is β-arrestin1 (Mo et al., 2008). The β-arrestins are two ubiquitous proteins that are best known for their role as cytoplasmic adapters in the regulation of G protein-coupled receptors and other signaling molecules (DeWire et al., 2007). Additional functions for β-arrestins in the nucleus have also been described (Kang et al., 2005). In line with this reasoning, Mo et al. propose a model whereby β-arrestin1, but not β-arrestin2, promotes the dephosphorylation of nuclear STAT1 by acting as a scaffold to directly facilitate recruitment of phosphatase TC45. This made β-arrestin1 an interesting object for our studies of STAT1 dimer reorientation and its effects on dephosphorylation. Here we present the results of our experiments, which contrary to expectations provide evidence against the reported negative-regulatory role of β-arrestin1 in STAT1 signaling.

## Results and Discussion

### Overexpression of β-Arrestin1 Does Not Diminish STAT1-Dependent Reporter Gene Activity

**Figure 1:**
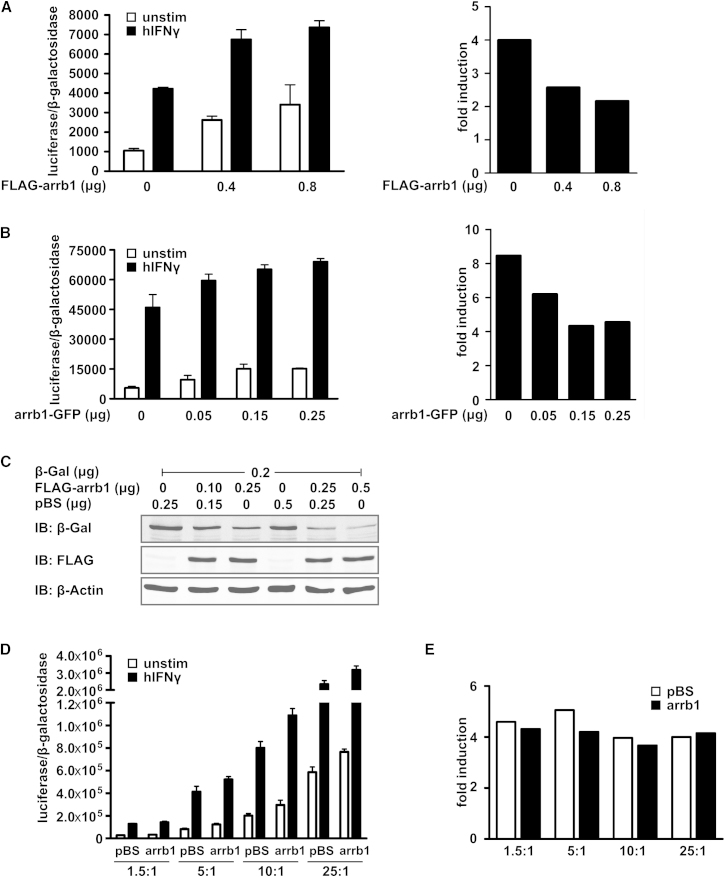
Cotransfection of β-Arrestin1 Has Nonspecific Inhibitory Effects on Gene Transcription (A and B) Interferon-γ-induced luciferase reporter activity in untreated and IFNγ-treated HeLa cells using increasing amounts of two β-arrestin1 constructs as indicated. Error bars represent means ± standard deviation for one representative experiment done in triplicates. The respective values for “fold induction” (induced/uninduced) are shown in the panels on the right. (C) Immunoblot showing the effects of FLAG-tagged β-arrestin1 on coexpressed β-galactosidase in HeLa cells. After western blotting, the nitrocellulose membrane was cut horizontally at the ∼80 kDa position. The upper membrane was probed with antibody against β-galactosidase; the other was consecutively probed with anti-FLAG-tag and anti-β-actin. (D and E) Interferon-γ-induced luciferase reporter activity in untreated and IFNγ-treated HeLa cells. The cells were transfected with plasmid pBS (control) or the expression plasmid for GFP-tagged β-arrestin1, and in addition with different ratios of plasmids encoding β-galactosidase and the luciferase reporter gene as indicated. Error bars represent means ± standard deviation of one experiment done in triplicate. The respective values for “fold induction” (induced/uninduced) are shown in (E).

At first we wanted to confirm that overexpression of β-arrestin1 diminishes IFNγ-induced transcription of a STAT1-dependent reporter gene in HeLa cells. We used C-terminally green fluorescent protein (GFP)-tagged human β-arrestin1 and N-terminally FLAG-tagged rat β-arrestin1, which in agreement with its evolutionary conservation can supplant functions and interactions of the human homolog (Scott et al., 2006; DeWire et al., 2007). Both constructs were overexpressed in HeLa cells but did not diminish IFNγ-induced reporter gene activity (Figures 1A and 1B). We noted that increased β-arrestin1 transfection led to an apparent rise in both constitutive and induced reporter gene activity, which overproportionally affected the former. Consequently, when depicted as “fold induction” ([induced transcription]/[constitutive transcription]), as done by Mo et al. (2008), the transcriptional activity indeed appeared to drop with increased β-arrestin1 levels (Figures 1A and 1B). We identified reduced expression of the β-galactosidase control gene as a cause (Figure 1C), probably due to titration of limiting coactivators by the cotransfected β-arrestin expression vectors. We therefore varied the reporter gene to control gene ratio between 1.5:1 and 25:1 to test whether a different transfection protocol could reveal the reported inhibitory effect of β-arrestin1 on STAT1-mediated gene induction (Figure 1D). However, interferon-inducible reporter gene activity was the same for all reporter-to-control-gene ratios used, and an inhibitory effect of β-arrestin1 was not recognizable (Figure 1E). We concluded that reduced STAT1 reporter gene transcription depicted as “fold induction” was likely due to an error associated with the cotransfection of β-arrestin1 expression vectors. We were unable to obtain the published β-arrestin1-encoding vectors from the authors upon request. It was hence not possible for us to rule out a similar error for their published data.

### STAT1 Dephosphorylation Is Unaltered in the Presence or Absence of β-Arrestin1

**Figure 2:**
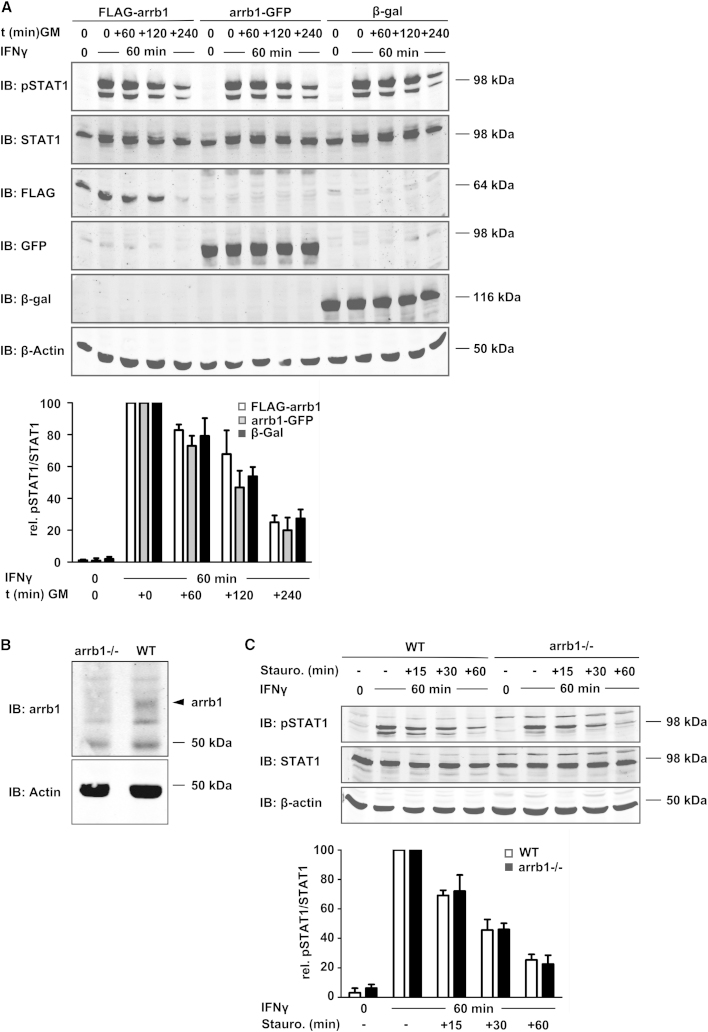
β-Arrestin1 Does Not Affect STAT1 Phosphorylation (A) Immunoblot showing a time course of STAT1 phosphorylation in IFNγ-treated HeLa cells that transiently express β-arrestin1 or β-galactosidase as indicated. Cells were left untreated or treated with IFNγ for 60 min, followed by incubation for 0–240 min in growth medium (GM) without interferon. Probing was done first with anti-Tyr701-phoshorylated STAT1 antibody, then with a mixture of anti-human STAT1 and anti-β-actin antibody, followed by a mixture of anti-GFP and anti-FLAG-tag, and finally with anti-β-galactosidase. Molecular weight marker positions are indicated. The diagram combines the results of this and another experiment. It shows specific Tyr701-phosphorylation of STAT1 under the different experimental conditions (specific Tyr701 phosphorylation after 60 min IFNγ was set as 100). Results were calculated as the mean ± standard deviation. (B) Shown are immunoblot analyses of wild-type (WT) and β-arrestin1-deficient MEFs using anti-β-arrestin1 antibody. Reprobing was done with anti-β-actin. Positions of molecular weight markers and β-arrestin 1 are indicated. (C) Top, immunoblot analyses of STAT1 dephosphorylation in WT and mutant MEFs. Bottom, diagram combining the results of this and two additional experiments giving specific Tyr701 phosphorylation of STAT1. The value for 60 min IFNγ was set as 100. Results were calculated as the mean ± standard deviation.

Next we assessed the reported acceleration of STAT1 dephosphorylation by β-arrestin1. Based on the published work, we expected that overexpression of β-arrestin1 in HeLa cells would diminish tyrosine phosphorylation of the endogenous STAT1. However, this was not the case, since STAT1 phosphorylation kinetics was identical under all conditions examined regardless of the presence of increased β-arrestin1 (Figure 2A). To avoid the limitations of transfection assays, we obtained mouse embryonic fibroblasts (MEFs) deficient in β-arrestin1 and control fibroblasts (Kohout et al., 2001). First, we used a commercially available antibody to confirm β-arrestin1 expression in wild-type but not the β-arrestin1-deficient MEF (Figure 2B). We then studied the tyrosine dephosphorylation of STAT1 in the wild-type cells and the β-arrestin1-deficient cells (Figure 2C). This was done by treating the cells for 60 min with IFNγ before adding the protein kinase inhibitor staurosporine for up to 60 min to terminate abruptly the ongoing protein phosphorylation. As expected, after 1 hr in the presence of staurosporine the concentration of tyrosine-phosphorylated STAT1 had fallen by ∼80% in the wild-type cells. Contrary to the previous report, however, STAT1 dephosphorylation was not diminished in the cells lacking β-arrestin1 (Figure 2C). Thus, STAT1 activation kinetics was unchanged regardless of whether β-arrestin1 was overexpressed or deleted.

### Reduced Rather Than Enhanced STAT1 Transcription Activity in β-Arrestin1-Deficient Cells

**Figure 3:**
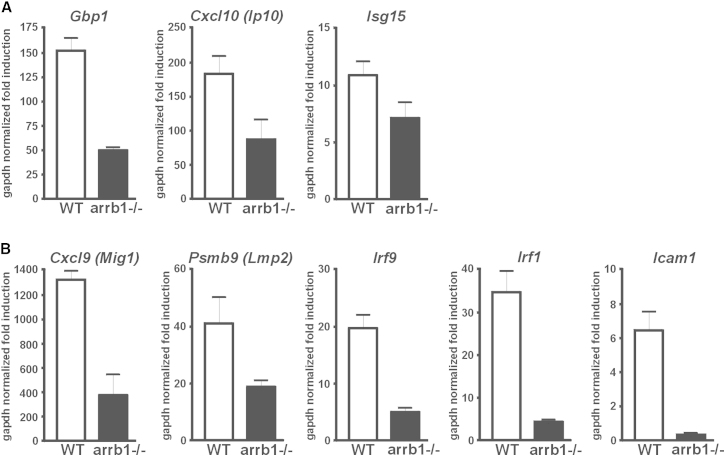
β-Arrestin1 Does Not Inhibit IFNγ Signaling (A and B) Quantitative RT-PCR analyses of the relative mRNA expression of endogenous genes in WT and β-arrestin1-deficient MEFs after IFNγ stimulation for 6 hr. Data are the mean ± standard deviation of three independent experiments.

We then used β-arrestin1-deficient MEFs in conjunction with the primer sequences and qPCR conditions of Mo et al. (2008) to determine IFNγ-induced expression of *Gbp1*, *Cxcl10*, and *Isg15* genes. According to their work interferon-induced transcription of these genes is 5- to 25-fold higher for β-arrestin1-deficient MEFs than for wild-type cells. In our hands, in contrast, lack of β-arrestin1 did not result in increased transcription. In fact, transcription was impaired in the β-arrestin1-deficient cells, an observation we also made for the five IFNγ-inducible genes we tested in addition (Figure 3). It remains to be determined if β-arrestin1 has a STAT1-independent stimulatory role specifically in interferon signaling, or whether the observed effects on gene transcription rather are a reflection of β-arrestin1’s involvement in a multitude of cellular functions. Irrespective of that, our results contrast with those of Mo et al. We note that Mo et al. use MEFs deficient in both β-arrestin1 and β-arrestin2. These cells are transiently transfected to reconstitute β-arrestin1 expression (Mo et al., 2008). We question the appropriateness of this experimental setup to discern the biological activities of individual β-arrestins. For example, the authors presume comparable β-arrestin1 expression in wild-type cells and their reconstituted MEFs based on western blotting results (see their Figure 4C). Yet this experiment does not fully clarify the issue in the absence of information about transfection efficiencies and hence cellular expression levels. If, however, the authors’ presumption is indeed correct, the problem arises that gene expression is upregulated 5- to 25-fold in the arrestin1/2 double-deficient MEFs, whereas reconstitution with β-arrestin1 merely halves gene expression according to Mo et al. This discrepancy indicates that β-arrestin double knockout MEFs do not revert to wild-type characteristics with regard to STAT1 signaling upon transfection of β-arrestin1. As this limits the value of β-arrestion1/2 doubly deficient cells for studying the effects specifically of β-arrestin1 on STAT1, we have instead used cells deficient solely in β-arrestin1.

### Evidence against β-Arrestin1 as a STAT1-Interacting Protein

**Figure 4:**
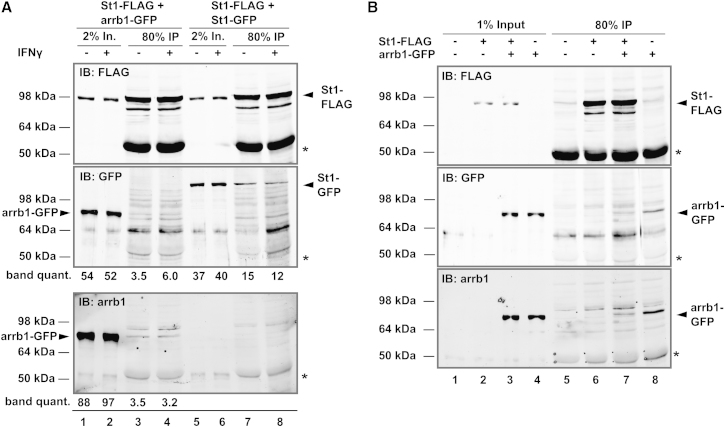
Coimmunoprecipitation Experiments Do Not Confirm STAT1 as a β-Arrestin1-Interacting Molecule (A) Immunoprecipitations using anti-FLAG-tag antibody and extracts from untreated and IFNγ-treated HEK293T cells coexpressing FLAG-tagged STAT1 and β-arrestin1 (arrb1-GFP) or STAT1 (STAT1-GFP). Shown are inputs (In.) and immunoprecipitates (IP). Immunoblotting was done consecutively with anti-GFP, anti-FLAG-tag, and anti-β-arrestin1 antibodies. Molecular weight marker positions are indicated on the left, and arrowheads point to overexpressed proteins. Asterisks indicate immunoglobulin heavy chains. The intensity (arbitrary light units) of arrb1-GFP and STAT1-GFP bands, corrected for background, is reported below the middle and bottom panels where feasible. (B) Shown are immunoprecipitations as in (A) using extracts from HEK293T cells coexpressing FLAG-tagged STAT1 and β-arrestin1 (arrb1-GFP) in the combinations indicated. Prior to antibody incubation, the membrane was stained with Ponceau S to reveal immunoglobulin heavy-chain bands, marked with (^∗^). In lanes 5–8, their upper and lower boundaries were marked with needle pinches, which remain visible in the immunoblots.

In the final set of experiments, we evaluated β-arrestin1 as a STAT1-interacting protein. FLAG-tagged STAT1 and GFP-tagged β-arrestin1 were expressed in HEK293T cells either alone or together. The interaction of β-arrestin1 and STAT1, which was reported to be maximal in IFNγ-treated cells, was then analyzed by anti-FLAG immunoprecipitation. Alternatively, STAT1-FLAG was coexpressed with GFP-tagged STAT1 as a positive control since STAT1 forms equally stable homodimers before and after stimulation with IFNγ (Wenta et al., 2008). As shown in Figure 4A, top panel, probing with anti-FLAG antibody of extracts from cells cotransfected with FLAG-tagged STAT1 and GFP-tagged-β-arrestin1 (lanes 1 and 2) or GFP-tagged STAT1 (lanes 5 and 6), respectively, showed an ∼98 kDa band expected for FLAG-tagged STAT1. Reprobing with anti-GFP antibody (middle panel) revealed a band of ∼80 kDa band in lanes 1 and 2 expected for GFP-tagged β-arrestin1; and a band of ∼110 kDa in lanes 5 and 6 expected for GFP-tagged STAT1. Probing with anti-β-arrestin1 antibody (bottom panel) labeled an ∼80 kDa band in lanes 1 and 2, but not 5 and 6, confirming β-arrestin1 overexpression. These results demonstrated that both STAT1 and β-arrestin1 were well-expressed in the cotransfected cells. When the material that coprecipitated with FLAG-tagged STAT1 was probed with anti-GFP to detect coprecipitating β-arrestin1-GFP, shown in the middle panel, lanes 3 and 4, a band that comigrated with GFP-tagged-β-arrestin1 was labeled, albeit multiple unrelated bands were stained more intensely. Reprobing with anti-β-arrestin1 antibody nonetheless confirmed this band to contain β-arrestin1 (bottom panel). Since the same antibody was used for detection of β-arrestin1 and STAT1, namely anti-GFP, it was possible to provide also a quantitative assessment for the efficiency with which these two proteins coprecipitated with the FLAG-tagged STAT1. The quantitative comparison, which was done after normalization of the signals for precipitated β-arrestin1 and STAT1 using their respective inputs, demonstrated that the interaction of STAT1 with itself was three to six times stronger than with β-arrestin1. In our experiments the signal intensities for coprecipitating β-arrestin1 were not only weak but moreover did not increase when extracts from IFNγ-treated cells were used (middle and bottom panels, compare lanes 3 and 4). As an additional control for the specificity of the observed interaction presumably between β-arrestin1 and STAT1, we performed immunoprecipitations using anti-FLAG-tag antibody in the absence and presence of its antigen, namely FLAG-tagged STAT1 (Figure 4B). As seen in the previous experiments, anti-FLAG-tag immunoprecipitations with extracts that contained both β-arrestin1 and the FLAG-tagged STAT1 gave a weak β-arrestin1 signal (middle and bottom panels, lane 7). This signal was expectedly missing when β-arrestin1 was missing in the extracts (middle and bottom panels, compare lane 7 to lane 6). However, the converse experiment using extracts devoid of FLAG-tagged STAT1 did not result in absent or at least diminished precipitation of β-arrestin1 (middle and bottom panels, compare lanes 7 and 8). We therefore concluded that the observed precipitation of β-arrestin1 was not due to interactions specifically with STAT1.

STAT1 activation is indispensable for the execution of interferon-γ functions such as antimicrobial protection. Interacting proteins that can reduce the activity of STAT1 are therefore of pharmaceutical interest and potentially of clinical relevance, as was suggested for β-arrestin1 by Mo et al. (2008). However, our experiments did not identify β-arrestin1 as a STAT1-interacting protein, and they showed that β-arrestin1 did not reduce the activation of STAT1. We accordingly found that the transcription of interferon-γ target genes was not inhibited in the presence of β-arrestin1. In summary, our data do not support a negative-regulatory role for β-arrestin1 in interferon-γ signaling.

## Experimental Procedures

### Cell Culture, Transfections, Plasmids, and Cytokines

Immortalized MEFs deficient in *β-arrestin1* and wild-type control cells were provided by Dr. R.J. Lefkowitz, Duke University. HEK293T, HeLa, and MEF cells were kept in growth medium, DMEM supplemented with 10% FBS and 1% penicillin/streptomycin, in a humidified incubator with 5% CO_2_ at 37°C. Cells grown to 70%–90% confluence were transfected using Lipofectamine according to the manufacturer’s recommendations (Invitrogen), followed by subsequent experimentation after 24 hr. Plasmids encoding human β-arrestin1-GFP (arrb1-GFP) and rat FLAG-β-arrestin1 (FLAG-arrb1) were provided by Dr. N. Holliday, University of Nottingham, and Dr. S. Marullo, Institut Cochin, Paris, respectively. The interferon-γ-dependent luciferase reporter gene 3xLy6E GAS-LUC containing a triple STAT1 binding site was described (Wen et al., 1995). Plasmid pSV-βgal, a β-galactosidase expression plasmid, was purchased from Promega. Human (#407306) and mouse IFNγ (#407303) were obtained from Merck Millipore.

### Reporter Gene Assays and Associated Controls

HeLa cells were transfected on 24-well plates with 0.25 μg/well plasmid encoding β-galactosidase, 0.25 μg/well 3xLy6E GAS-LUC reporter gene, and the indicated amounts of plasmid encoding β-arrestin1 tagged with either GFP or FLAG. The amount of transfected DNA was kept constant by adding plasmid pBluescript (pBS). After 24 hr, the cells were left untreated or treated for 6 hr with 50 U/ml human IFNγ, followed by cell lysis in buffer containing 25 mM glycylglycine, 15 mM MgSO_4_, 4 mM EGTA, 1% Triton X-100, 1 mM DTT (pH 7.8). Luciferase activity was determined with Luciferase-Assay-System (Promega) according to the manufacturer’s instructions. β-galactosidase activity was photometrically measured at room temperature at a wavelength of 405 nm after incubation (30–60 min) of the extracts (5 μl) in 280 μl buffer containing 0.75 mM NaH_2_PO_4_/Na_2_HPO (pH 7.4), 1 mM MgCl_2_, 1 mg/ml o-nitrophenyl-β-D-galactoside. The enzymatic reaction was stopped by adding 0.5 M Na_2_CO_3_. The readings were used for normalization of transfection efficiency. Control experiments (Figure 1D) with varying ratios of luciferase and β-galactosidase expression plasmids were done with HeLa cells under the conditions described before. The [luciferase:βgal] ratios stated were obtained by using the following combinations of plasmid 3xLy6E GAS-LUC and pSV-βgal: [*1.5:1*] 0.3 and 0.2 μg/well, [*5:1*] 0.42 and 0.08 μg/well, [*10:1*] 0.495 and 0.05 μg/well, and [*25:1*] 0.5 and 0.02 μg/well. In addition, a fixed amount (0.25 μg/well) of plasmid encoding β-arrestin1 (arrb1-GFP) or pBS was included as indicated. The control experiments of Figure 1C were done with HeLa cells on 12-well plates cotransfected with 0.2 μg/well of plasmid pSV-βgal and the indicated amounts per well of FLAG-arrb1. The total amount of transfected DNA was kept constant by adding pBS as indicated.

### STAT1 Tyrosine Phosphorylation/Dephosphorylation Assays

STAT1 phosphorylation as shown in Figure 2A was determined using HeLa cells on 6-well plates transfected with 0.8 μg/well of the indicated vectors expressing β-arrestin1 or β-galactosidase as the control. Twenty-four hours later the cells were passaged (dilution 1:2.5), and after a further 24 hr the cells were left untreated or treated for 1 hr with 50 U/ml human IFNγ, followed by a medium change and incubation with IFN-free growth medium for the indicated time periods. STAT1 dephosphorylation (Figure 2C) was detected using wild-type and β-arrestin1-deficient MEFs left untreated or treated with 50 U/ml mouse IFNγ for 1 hr. Subsequently, 0.5 μM tyrosine kinase inhibitor staurosporine (#569397, Calbiochem) was added to the cells for the indicated lengths of time, followed by cell lysis in boiling SDS sample buffer. The lysates were resolved by SDS-PAGE and western blotted.

### Whole-Cell Extractions, SDS-Page, Western Blotting, and Antibodies

Cells were extracted on ice for 30 min in buffer containing 2 mM EGTA, 0.2 mM EDTA, 1 mM Na-Vanadate, 50 mM NaF, 280 mM NaCl, 0.5% (v/v) NP-40, 10% (v/v) glycerol, 1 mM DTT, protease inhibitors PMSF (0.54 mM) and Complete (Roche), and 50 mM Tris/HCl (pH 7.4). Soluble whole-cell extracts were resolved by SDS-PAGE and were analyzed by quantitative western blotting using a Li-Cor Odyssey system as described (Droescher et al., 2011). Anti-mouse STAT1α (sc-591), anti-human STAT1α (sc-345), anti-GFP (sc-8334), and anti-β-arrestin1 (sc-9182) were purchased from Santa Cruz Biotechnology. Anti-β-actin (A5441) and anti-FLAG M2 (F3165) were from Sigma. Anti-phospho-Tyr701 STAT1 (#9171) was from Cell Signaling Technology; anti-β-galactosidase (A11132) was from Molecular Probes. IRdye800CW-conjugated anti-mouse (#926-32212) and anti-rabbit (#926-32213) IgG secondary antibodies were purchased from Li-Cor Bioscience.

### Immunoprecipitation

HEK293T cells grown on 10 cm plates were transfected with 2.5 μg each of plasmids encoding STAT1-FLAG and β-arrestin-GFP or STAT1-GFP. Twenty-four hours later cell cultures were passaged (dilution 1:2), and after a further 24 hr the cells were left untreated or treated for 1 hr with 50 U/ml IFNγ. Soluble whole-cell extracts, containing 500 μg protein as determined by Bradford assay (Biorad), were rotated at 4°C with 5 μl anti-FLAG M2 for 90 min before adding 12 μl (settled volume) protein A/G PLUS agarose (sc-2003, Santa Cruz) and rotating for another 2 hr. After centrifugation (1,000 g) at 4°C for 4 min, the precipitate was washed twice with whole-cell extraction buffer and eluted in SDS sample buffer for western blotting.

### qRT-PCR

MEF cells were grown overnight in serum-depleted growth medium (1% (v/v) FBS), followed by incubation in growth medium with or without 100 U/ml mouse IFNγ. Subsequently, total RNA was extracted, followed by reverse transcription, and expression of selected genes was measured by qPCR using SYBR Green. The primers used for *Gbp1*, *Cxcl10*, and *Isg15* were as described by Mo et al. (2008). Expression of *Psmb9* was determined with primer pair 5′-CGGGGGTGTCGTGGTGGGCTCTG and 5′-CGCCGGCACTCCTCAGGGGTCAT (reverse), and that of *Irf9* with 5′-GCCTTTGCCCCATCCCCATCTC and 5′-CCCCTGGCCCTGGAAGTACTGG (reverse). For further experimental details and the primers for *Cxcl9*, *Irf1*, and *Icam1*, see Begitt et al. (2011).
